# Exploring the role of diet quality and adiposity in the pain experience: a mediation analysis

**DOI:** 10.1007/s00394-025-03772-0

**Published:** 2025-08-23

**Authors:** Susan J. Ward, Alison M. Coates, Sharayah Carter, Katherine L. Baldock, Ty E. Stanford, Carolyn Berryman, Tasha R. Stanton, Jonathan D. Buckley, Alison M. Hill

**Affiliations:** 1 Alliance for Research in Exercise, Nutrition and Activity (ARENA), University of South Australia, Adelaide, South Australia Australia; 2 Clinical and Health Sciences, University of South Australia, Adelaide, South Australia Australia; 3 Allied Health and Human Performance, University of South Australia, Adelaide, South Australia Australia; 4 School of Health and Biomedical Sciences, Royal Melbourne Institute of Technology (RMIT University), Melbourne, VIC Australia; 5 Innovation, IMPlementation and Clinical Translation (IIMPACT), University of South Australia, Adelaide, South Australia Australia; 6 Persistent Pain Research Group, Hopwood Centre for Neurobiology, South Australian Health and Medical Research Institute (SAHMRI), Adelaide, South Australia Australia

**Keywords:** Diet quality, Weight loss, Chronic musculoskeletal pain, Adiposity, Mediation analysis

## Abstract

**Purpose:**

Improving diet quality may lower chronic musculoskeletal pain (CMP) directly or through weight loss. This study examined whether a dietary intervention for weight-loss improved diet quality and CMP in adults with elevated adiposity. It also investigated whether adiposity mediated a relationship between diet quality and pain.

**Methods:**

This secondary analysis of data from another study included 104 Australian adults (25–65 years) with overweight/obesity (BMI, 27.5–34.9 kg/m^2^) who completed a 3-month dietary intervention targeting 30% energy restriction. Baseline and 3-month measures included diet quality (Dietary Guideline Index (DGI)), presence of CMP, pain related quality-of-life (Short-Form-36 bodily pain scale (SF36-BPS)), pain severity (McGill Pain Questionnaire (MPQ)) and adiposity (weight, waist circumference (WC), percent body fat (BF)). Linear mixed models estimated the effect of the dietary intervention on these outcomes. Structural equation modelling estimated the direct effects of changes in diet quality on CMP, and proportion mediated by changes in adiposity.

**Results:**

Participants improved diet quality (DGI total score) by 22% (p < 0.001) and achieved weight loss (− 7.1 ± 0.3 kg, 95% CI − 7.7, − 6.4). Presence of CMP reduced from 50 to 24% (p < 0.001). Pain-related quality of life improved, and pain severity lessened. Reductions in weight, WC, or BF did not mediate improvements in pain characteristics. Improved diet quality (∆DGI) was directly associated with lower pain severity (∆MPQ), accounting for reductions in the mediator, WC (β = − 0.085, 95% CI − 0.151, − 0.019) and BF (β = − 0.073, 95% CI − 0.135, − 0.012).

**Conclusion:**

On average, diet quality improved and pain lessened following a 3-month dietary intervention for weight-loss. Changes in adiposity did not mediate this relationship.

**Supplementary Information:**

The online version contains supplementary material available at 10.1007/s00394-025-03772-0.

## Introduction

The prevalence of overweight and obesity continues to increase globally making it a major public health concern [1, 2]. Elevated adiposity is associated with an increased risk for, or exacerbation of, a wide spectrum of health conditions including musculoskeletal disorders, which are a leading contributor to the global burden of disease [3, 4].

Accordingly, the body of evidence for the coexistence of overweight/obesity and chronic musculoskeletal pain is growing [5, 6]. Excess weight places stress on joints and tissues, altering biomechanics and leading to physical limitations that impair movement [7, 8]. These restrictions further impact energy balance and weight status [9, 10]. In addition, having a higher fat mass is linked to an increased risk for developing pain in the lower back, knee, foot, and ankle, which is often experienced as multi-site pain [11–16]. Adipose tissue itself is metabolically active, producing and releasing proinflammatory cytokines, which contribute to low-grade systemic inflammation [17, 18], and may play a role in the development of chronic pain [19–21].

Most reviews and meta-analyses recognise the effectiveness of weight loss interventions (behavioural, pharmaceutical, or surgical) in reducing musculoskeletal pain [22–24]. However, included studies primarily focus on mechanical loading and pain related to knee and hip osteoarthritis (OA) [22–24]. In addition to decreasing the mechanical load on joints, weight loss can also lessen systemic low-grade inflammation by reducing fat mass [25]. Nevertheless, weight loss and significant reductions in joint pain are not reported in all meta-analyses [23, 26] and factors beyond weight loss may play a role in improving pain.

Energy restriction is a key component of weight loss interventions, yet this is often achieved through detrimental dietary practices or patterns that restrict foods or nutrients, and lack evidence for sustained weight loss [27]. The evaluation of diet quality is important, not only because improved diet quality can aid weight loss [28], but a high-quality diet may confer health benefits independent of weight loss [29, 30].

Dietary patterns that promote health and reduce risk of chronic disease are defined in evidence-based guidelines [31, 32]. Consequently, assessment of adherence to these guidelines through a priori diet quality indices allow associations with health outcomes to be made [31, 33]. Independent of weight status, evidence from dietary interventions support higher diet quality being associated with reductions in non-cancer chronic pain [34, 35]. Most recently, a systematic review of 14 cross-sectional and 6 longitudinal studies generally supported an association between healthful a priori dietary patterns and non-cancer pain [36]. However, the establishment of causality was limited by inconsistent findings and poorly defined methodology from included longitudinal studies [36].

While nutrient-rich dietary patterns are recommended for the management of chronic pain [37, 38], it remains uncertain whether dietary interventions targeted at improving diet quality directly affect pain, or if they influence pain indirectly via intermediary factors such as changes in adiposity. Previous research, including our own [39], has focused on the impact of weight loss (and subsequent adiposity reduction) on pain in adults with overweight and obesity. However, the role of diet quality in this relationship has been less extensively studied.

**Fig. 1:**
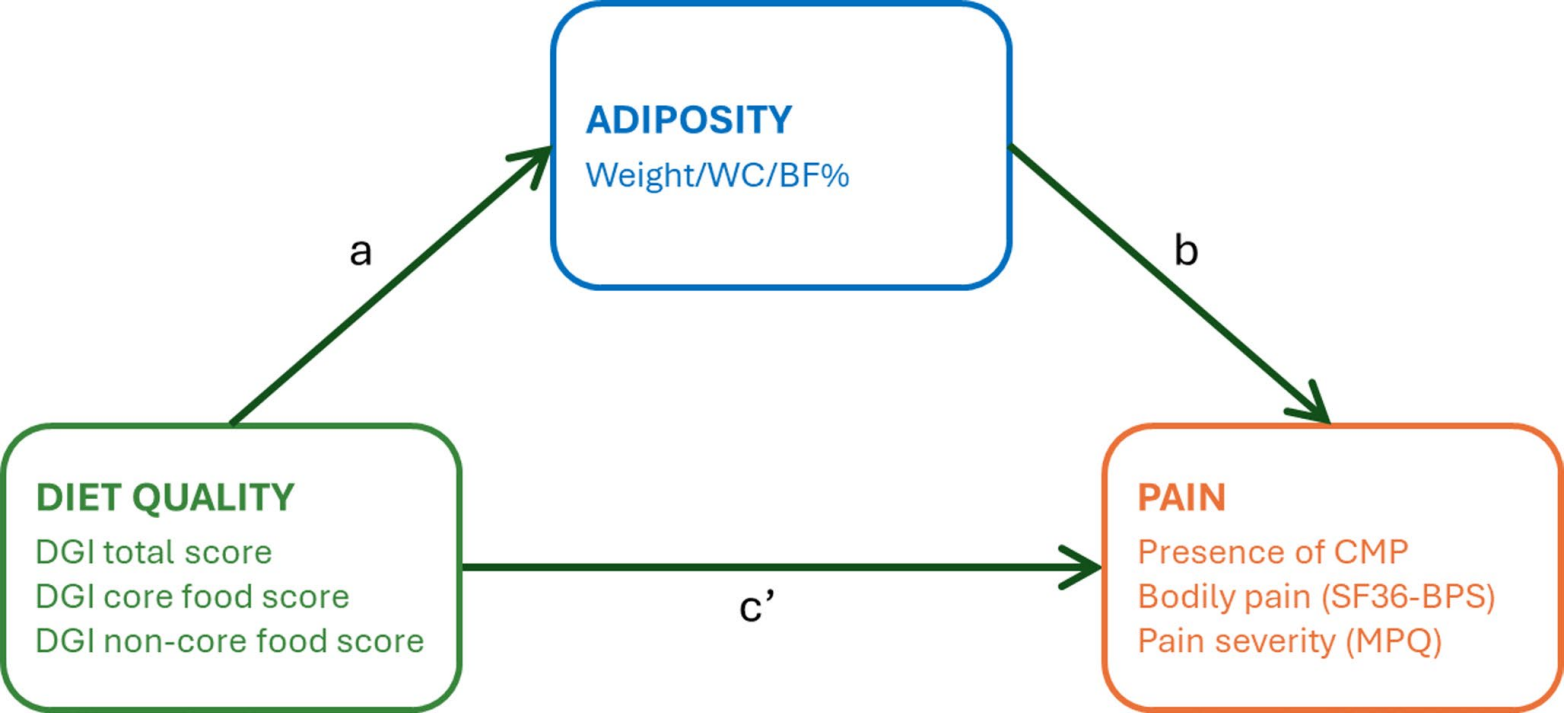
Mediation analysis model of associations between diet quality and pain outcomes, mediated by adiposity. **Path a** represents the regression coefficient for the relationship between diet quality and adiposity. **Path b** represents the regression coefficient for the association between adiposity with pain. Mediation paths are decomposed into (1) the indirect effect (path a * b), which denotes the relationship between diet quality and pain through the mediator (i.e., effect mediated by adiposity), and (2) the direct effect (**path c’**), the relationship between diet quality and pain, not through the mediator (i.e., effect not explained by adiposity). BF%, percent body fat; CMP, chronic musculoskeletal pain; DGI, dietary guideline index; MPQ, McGill Pain Questionnaire; SF36-BPS, Short-Form-36 bodily pain scale; WC, waist circumference

In mediation analyses, a mediator is an intermediate variable that helps explain the mechanism by which an independent variable influences an outcome, thereby helping to unravel the causal pathways by which the intervention achieves its effect. In this study, mediation analyses were used to evaluate the interrelationships between diet, pain and adiposity. We hypothesised that (1) an energy-restricted diet will improve diet quality, reduce adiposity, and alleviate pain characteristics in individuals with overweight or obesity, and (2) adiposity (weight, waist circumference, body fat) will mediate the relationship between diet quality and pain in individuals with excess weight, both at baseline and following an energy-restricted diet intervention. A schematic of the mediation model is presented in Fig. 1.

## Methods

### Participants and study design

This was a secondary analysis of data from a dietary-induced weight-loss intervention conducted at the University of South Australia (January 2019–March 2021). The primary study was registered on the Australian New Zealand Clinical Trials Registry, ACTRN12618001861246. A detailed protocol, including participant eligibility [40], and a primary outcomes paper have both been published [41]. Briefly, adult volunteers (aged 25–65 years, BMI 27.5–34.9 kg/m^2^) were recruited from Adelaide and surrounding areas for a 9-month (3-month weight loss period followed by 6-months weight maintenance) parallel-arm, randomised controlled trial. The BMI range allowed for sufficient weight loss while minimising the potential risk of confounding by chronic health conditions, which become more prevalent as BMI increases [41]. All participants followed a 30% energy-restricted diet aligned with the Australian Guide to Healthy Eating [42]. This included 15% of energy intake from either raw unsalted almonds or isocaloric carbohydrate-rich snacks (fruit cereal bar and rice crackers) in an otherwise nut-free diet, to test the primary hypothesis that almond consumption would lead to greater weight loss and limit weight regain compared with an energy-matched nut-free diet [41]. For this study, we were interested in the overall effects of the energy-restricted dietary modification during the 3-month weight loss phase on health-related quality of life and pain outcomes, therefore data from the almond and nut-free groups were combined. The diets were designed to achieve a 0.5–1.0 kg/week weight-loss, with individual energy requirements determined using the Schofield equation with an appropriate physical activity level [43]. Dietary counselling was provided by an Accredited Practicing Dietitian at baseline, then every two weeks during the 3-month weight loss period.

### Clinic visits

Participant’s dietary intake, anthropometric measures, fasting venous blood samples (minimum 10 h, with no alcohol in previous 24 h), and pain measures were performed at the University of South Australia’s Clinical Trials Facility at baseline and 3-months, noting that some of the 3-month clinic visits were interrupted by South Australian Government enforced Covid-19 restrictions from April-June 2020. During this time, body mass was measured at home using Bluetooth-enabled scales (Withings/Nokia WBS06, Nokia), but anthropometric data (waist circumference, body composition) were not obtained. Weight measurements from Bluetooth-enable scales were included in analysis on confirmation there was no statistically significant difference in the magnitude of weight loss achieved by participants whose weight was captured using these scales compared to participants whose weight was measured in clinic.

## Measures

### Exposure—dietary intake and diet quality

Dietary intake was captured via 4-day weighed food records in the week preceding baseline and 3-month visits and analysed using FoodWorks Nutritional Analysis Software Version 10 (Xyris Software, Brisbane, Australia). Established cut offs (< 2090 or > 16,720 kJ/day, 500–4000 kcal) were applied to total energy intake estimates to exclude participants suspected of mis-reporting their dietary intake [44]. One participant with a reported energy intake falling below the lower cut-off was excluded.

Dietary data extracted from FoodWorks were used to analyse diet quality using an algorithm based on the Dietary Guideline Index (DGI) for weighed food records [45, 46]. The DGI uses age- and sex- specific dietary guidelines to score 10 food components. Total DGI scores ranged from 0 to 120, with higher scores reflecting better adherence to the 2013 Australian Dietary Guidelines and a higher quality diet [42]. DGI core food components, based on intake and variety from the Australian Dietary Guidelines core food groups (food variety, vegetables, fruit, grains, lean meats and alternatives, dairy and alternatives and fluid intake), were scored 0–70. Non-core food components were scored 0–50 and reflect foods that should be limited or consumed in moderation (including unsaturated spreads and oils, plus alcohol and foods high in added fats, sugar, sodium). Almonds were categorised in the lean meat and alternatives component. The rice crackers provided to the nut-free diet group contributed to grain serves whereas the oven-baked fruit cereal bar was a discretionary item. Supplementary Table S1 presents the components and scoring approach for this DGI.

### Outcome—pain

Pain measures used to assess the effect of intervention were presence of CMP, pain related quality of life, and changes in the nature and severity of pain.

Pain experienced in the preceding 24 h was captured on body charts. The location and number of pain sites, ranked from most to least troublesome, were documented and presence of CMP was defined where the duration of pain at any site extended ≥ 3 months [47].

The RAND Short Form-36 Health Survey bodily pain transformed scale (SF36-BPS) was used to assess pain related quality of life in all participants [48, 49]. This measure is derived from two items answered using a Likert scale assessing pain intensity and pain’s interference with daily activities. Scores from both items are summed to provide a raw score that is transformed to a 0–100 scale, with a higher score representing less bodily pain [49]. The SF36-BPS has demonstrated good levels of validity and reliability, with internal consistency [50].

The nature and severity of pain at each pain site was captured in participants reporting CMP via the Short Form McGill Pain Questionnaire (MPQ) [51]. Participants ranked 15 items (11 sensory and 4 affective words) on a scale from none (0), mild (1), moderate (2) to severe (3), to provide a summed pain severity score out of 45. The MPQ is considered acceptable, reliable, and valid for the evaluation of pain complaints and to measure the effects of interventions or pain relief in adults [50]. Pain severity scores were determined for both the pain site identified as the most troublesome and site-matched so that pain at the same site was compared (e.g., shoulder pain at both time points). To calculate differences from baseline in pain severity, an absence of CMP at 3-months was scored as 0 (change score calculated), whereas if only acute pain was reported at 3-months, this was not scored (change score not determined).

### Mediator—adiposity

Anthropometric measurements (height, weight, waist circumference), along with whole-body dual-energy X-ray absorptiometry (DEXA) scans (Lunar Prodigy Model, GE Healthcare, Wisconsin, USA) were conducted at clinic visits as previously described [40]. Higher weight, waist circumference, and BMI are indicators of higher adiposity. DEXA assessed body fat (BF) (determined using enCORE 2015 software (V.13.31)) is a measure of adiposity.

### Covariates

Demographic data captured at screening included participants’ age, sex, ethnicity, medical history including prescription medication and supplement use, and socio-economic status (Socio-Economic Indices for Areas (SEIFA) deciles of advantage and disadvantage) [52].

### Sample size

Sample size for this study relied on data available from the primary study [41]. The number of participants with complete diet, weight, and pain (presence of CMP and SF36-BPS) data determined the sample size at baseline (n = 134). Pre-post-intervention analyses were conducted in participants with complete diet, weight, and pain data at baseline and 3-months (n = 104). Due to the lack of a validated method for estimating necessary sample sizes or study power using the regression-based approach in mediation analyses, we were unable to provide such estimates [53, 54].

### Statistical analysis

Statistical analyses were undertaken using SPSS version 28.0 (SPSS, Chicago, IL, USA) and Stata Statistical Software Version 17 (College Station, TX). The level of significance was set as α = 0.05 for all analyses.

### Participant characteristics

Descriptive data are presented as means ± standard deviations (SD) for symmetrically distributed continuous variables, medians ± interquartile ranges (IQR) for skewed continuous variables and counts with percentages (%) for categorical variables.

### Effect of dietary intervention

Linear mixed effects models with participant random intercepts were employed to account for within-subject covariance in estimating the associated effects of the intervention on the outcomes diet quality, weight, body composition, inflammation, and pain. Time was included as a categorical fixed effect, denoting pre- and post-intervention timepoints; and models were adjusted for the fixed covariates of age, sex, baseline BMI. Estimated marginal means (EMMs) and associated standard errors (SE) are reported. A McNemar’s test was used to compare pre- and post-intervention differences in the proportion of participants reporting CMP.

## Mediation analyses

### Potential covariates

Baseline pair-wise associations between exposures, mediators, and outcomes (i.e. diet quality, adiposity, and pain) and potential confounders (age, sex, SEIFA, energy intake) were assessed to identify covariates for mediation analyses. Spearman-rank correlations were run for associations with age, SEIFA, and energy intake, while point serial correlations were run for sex. Chi-square test of independence used to examine associations between sex or SEIFA and dichotomised presence of CMP. Given most participants identified as Caucasian (84%), ethnicity was not included in the pair-wise associations analysis.

### Structural equation modelling

Structural Equation Modelling (SEM) mediation analyses were first conducted on data from baseline, to establish existing associations and additional potential mediating mechanisms, with DGI total score, DGI core and non-core food sub scores as exposures, and adiposity (weight, WC, and BF) as a mediator. Pain measures derived from SF36-BPS and MPQ were considered as continuous measures while presence of CMP was measured as a binary variable, therefore a generalised SEM (gsem), with a logistic link function, was required to link exogenous variables (predictors) to this endogenous variable (response). Model estimates were obtained using maximum likelihood, and bootstrapping was used to create empirical 95% confidence intervals as assumptions of symmetric sampling distributions of estimates under transform were not assured. The magnitude of mediation effect was estimated by calculating the ratio of indirect to total effect of the intervention (RIT). Baseline SEM mediation models included adjustment for age, sex, and baseline energy intake as potential mediator-outcome covariates.

Subsequently, we applied mediation analysis to the change data (3 months—baseline) to estimate the extent to which changes in the anthropometric measures (weight, WC, BF) after 3 months of energy restriction, were responsible for the association between changes in diet quality (DGI total, DGI core and non-core food sub scores) and changes in pain outcomes (presence of CMP, SF36-BPS and MPQ scores). Mediation analyses using change data were adjusted for age, sex, and the baseline values for each outcome.

## Results

### Participants

**Fig. 2:**
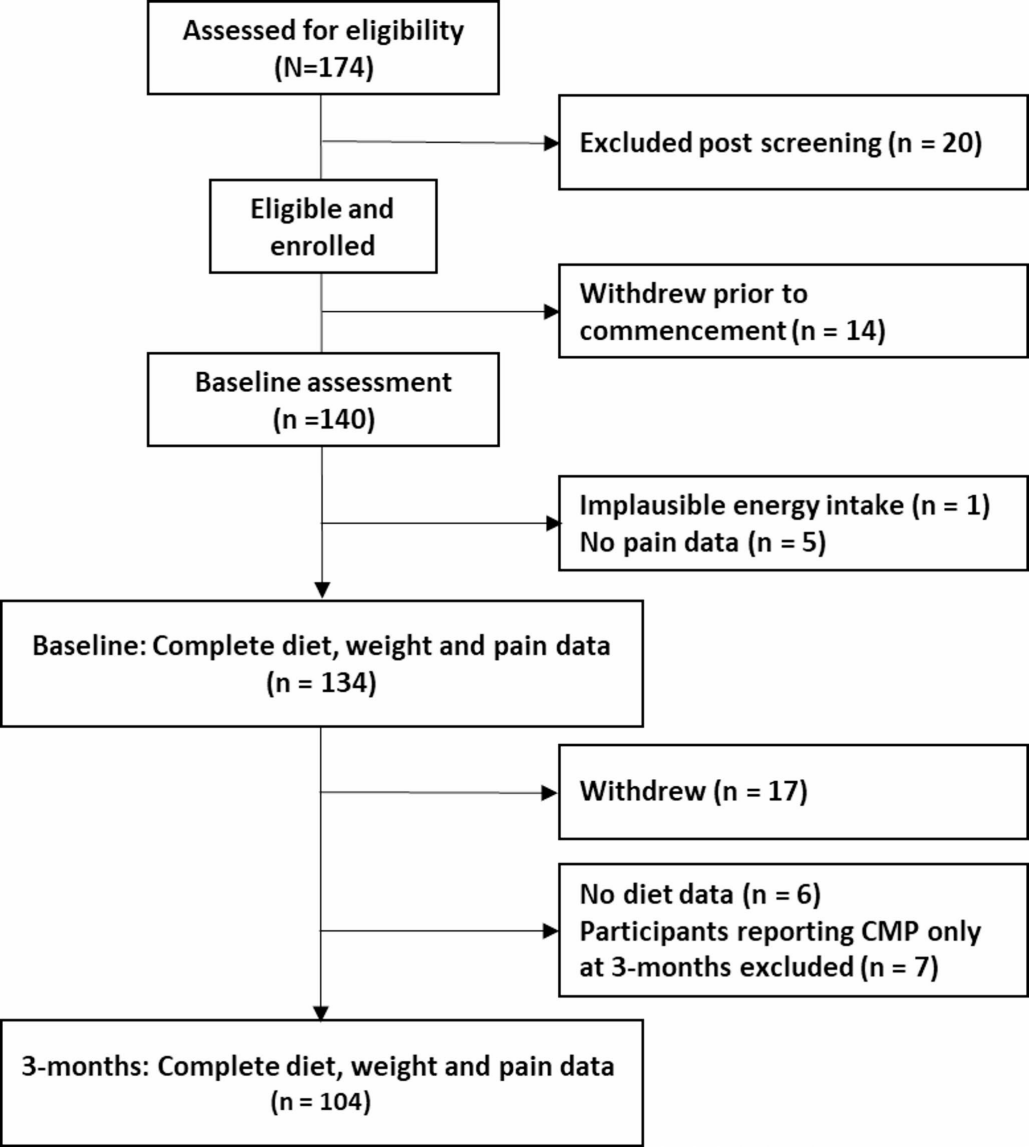
Flowchart displaying participant enrolment and inclusion in the analyses. CMP, chronic musculoskeletal pain

Of 174 people assessed for eligibility, 140 completed baseline assessments, with 6 excluded from baseline analyses due to implausible energy intake (n = 1) and missing pain data (n = 5). A further 30 participants were excluded from the final analyses due to withdrawal before 3-month assessment (n = 17), missing dietary intake data at 3-months (n = 6) and reporting of CMP only at 3-months (n = 7). Therefore, 104 participants had diet, weight and data on presence of CMP at both baseline and 3-months (Fig. 2).

### Participant characteristics (exposure, outcomes, and mediators)

Characteristics of participants with complete diet, weight, and pain data at baseline (n = 134), and following the intervention (n = 104) are presented in Table 1. Participants were mostly women (71%), with obesity (62% of participants), and identified as Caucasian (84%). Diet quality at baseline was poor, with average DGI total score below 50 (median score 45.0) out of maximal score of 120. Just under half the participants (n = 63, 47%) reported CMP at baseline; most participants with CMP reported single-site pain (n = 36, 57% of those reporting CMP). Spinal complaints (neck and back) accounted for 60% (n = 38) of CMP complaints followed by CMP in the lower limbs (n = 19, 30%) (data not shown). Mean SF36-BPS score was 71.8 ± 21.8, lower than the mean SF36-BPS Australian norms for SF36-BPS transformed percentage score (76.9 ± 25.0) [55]. On average, participants rated their pain via MPQ as “mild–moderate” [51].

**Table 1:** Baseline characteristics of the entire participant sample (n = 134) and of participants who completed the dietary intervention (n = 104)

Characteristic	Baseline participants (n = 134)	Dietary intervention (n = 104)
Women: men, n (%)^a^	95 (71): 39 (29)	73 (70): 31 (30)
Age (years)	47.7 ± 10.6	48.2 ± 10.7
SEIFA^b,c^	7.0 (3.0)	7.0 (4.0)
Energy intake (kJ/day)	9078.6 ± 2125.2	9120.9 ± 2012.8
*EXPOSURE*		
Diet quality DGI total score (0–120) DGI core food score (0–70) DGI non-core food score (0–50)	49.1 ± 15.5 35.5 ± 7.2 13.5 ± 13.1	49.5 ± 15.0 36.3 ± 6.8 13.2 ± 12.9
*OUTCOME(S)*		
Pain reported, n (%) Pain not CMP CMP reported	88 (65.7) 25 (18.7) 63 (47.0)	70 (67.3) 18 (17.3) 52 (50.0)
Pain related QOL SF36-BPS	71.8 ± 21.8	73.8 ± 20.6
Pain severity (MPQ) (0–45)^b,d^ Most troublesome pain site Matched pain site	6.0 (9.0) 5.0 (10.0)	5.5 (10.0) 5.0 (10.0)
*MEDIATOR(S)*		
Weight (kg)	87.8 ± 11.5	87.7 ± 11.6
BMI (kg/m^2^)	30.7 ± 2.2	30.6 ± 2.3
Waist circumference (cm)	101.9 ± 9.2	101.9 ± 9.3
Body composition (DEXA) Body Fat (%)	42.6 ± 6.2	42.7 ± 6.0

^a^ Relative number, n (%). Values are mean ± SD for normally distributed data. ^b^ Median (interquartile range) for skewed data. ^c^ n = 133(134), 103(104). ^d^ MPQ most troublesome site n = 63(134), 52 (104), MPQ site matched n = 45(134), 43(104)

BMI, body mass index; CMP, chronic musculoskeletal pain; DEXA, dual-energy X-ray absorptiometry; IQR, interquartile range; MPQ, McGill Pain Questionnaire; QOL, quality of life; SEIFA, Socio-Economic Indexes for Areas; SD, standard deviation; SF36-BPS; Short-form 36 bodily pain scale.

### Effect of intervention

At 3-months, participants had reduced their total daily energy intake from baseline (-3299 ± 176 kJ/day, 95% CI − 3648, − 2950), accompanied by reductions in weight (− 7.1 ± 0.3 kg, 95% CI − 7.7, − 6.4, 8%), WC (− 7.1 ± 0.5 cm, 95% CI − 8.2, − 6.1), and BF (− 3.9 ± 0.2%, 95% CI − 4.4, − 3.5).

Presence of CMP in participants reduced from 50% (n = 52) to 24% (n = 25) (p < 0.001), and SF36-BPS increased (+ 6.9 ± 2.1, 95% CI, 2.7, 11.1), reflecting lower levels of bodily pain. In participants reporting baseline CMP, there was a decrease in pain severity at the site identified as most troublesome as well as when identical pain sites were matched (∆MPQ -3.3 ± 0.8, 95% CI, − 5.0, − 1.7, and − 3.5 ± 0.9, 95% CI, − 5.3, − 1.8 respectively).

Table 2 presents the effect of the dietary intervention on diet quality. At 3-months, overall diet quality improved by 22% (DGI total score, + 26.0 ± 2.1, 95% CI 21.8, 30.2). Improvements were seen in DGI scores for intake of core foods (DGI core food score, + 3.9 ± 0.8, 95% CI 2.2, 5.6). Higher scores (indicating lower intake) for alcohol (+ 2.5 ± 0.4, 95% CI 1.6, 3.4) and discretionary foods (+ 19.6 ± 1.6, 95% CI 16.5, 22.7) contributed to the 44% improvement in DGI scores for non-core foods (+ 22.1 ± 1.6, 95% CI 18.7, 25.6).

**Table 2:** Model estimated marginal means (± standard error) in DGI component and subcomponent scores for participants completing the 3-month dietary intervention (n = 104). Covariates in the model; age, sex, and baseline BMI

Dietary guideline index (DGI) components (score range)	Baseline (EMM ± SE)	3-month (EMM ± SE)	P value
Energy intake (kJ/day)	9120.9 ± 174.4	5821.58 ± 176.0	< 0.001
1. Food variety (0–10)	3.6 ± 0.1	3.6 ± 0.1	0.869
2. Vegetables (0–10)	5.5 ± 0.2	7.0 ± 0.2	< 0.001
3. Fruit (0–10)	5.1 ± 0.3	5.9 ± 0.3	0.029
4. Grain (cereal) (0–5)	4.2 ± 0.1	3.2 ± 0.1	< 0.001
4a. Mostly wholegrain (0–5)	1.2 ± 0.2	2.3 ± 0.2	< 0.001
5. Lean meats & alternatives (0–10)	6.0 ± 0.2	7.5 ± 0.2	< 0.001
6. Dairy & alternatives (0–5)	3.0 ± 0.1	2.7 ± 0.1	0.093
6a. Reduced-fat dairy (0–5)	0.05 ± 0.1	1.0 ± 0.1	< 0.001
7. Fluid intake (0–5)	3.8 ± 0.1	3.4 ± 0.1	0.004
7a. Proportion water to total fluid (0–5)	3.9 ± 0.2	3.5 ± 0.2	0.051
Total core food components (0–70)	36.3 ± 0.8	40.2 ± 0.8	< 0.001
8. Moderate unsaturated spreads and oils (0–10)	2.0 ± 0.4	2.0 ± 0.4	1.000
9. Limit discretionary intake (0–30)	4.3 ± 1.1	23.9 ± 1.1	< 0.001
10. Limit alcohol (0–10)	6.8 ± 0.4	9.3 ± 0.4	< 0.001
Total non-core food components (0–50)	13.2 ± 1.3	35.3 ± 1.3	< 0.001
Total DGI (0–120)	49.5 ± 1.7	75.5 ± 1.7	< 0.001

BMI, body mass index; DGI, dietary guideline index; EMM, estimated marginal means; SE, standard error.

## Mediation

### Identification of SEM covariates

In determining which covariates to include in the model, we considered both statistical significance and theoretical relevance, aiming to account for key factors that could influence diet, adiposity and pain. Point serial correlations identified that being male was associated with higher weight and WC, and being female was associated with higher BF and higher DGI core food scores (Supplementary Table S2). Spearman-rank correlations indicated that higher energy intake was associated with higher DGI core scores, and with lower DGI non-core scores. Additionally, higher energy intake was positively associated with higher weight and WC. Age did not show any significant associations, except for a weak and non-significant trend with presence of CMP. Based on these findings, age, sex, and baseline energy intake were adjusted for in the baseline mediation models. Age, sex, and baseline values for each outcome were accounted for in the change mediation models.

### Baseline mediation outcomes

#### Adiposity as the mediator

Significant negative exposure-mediator relationships (path a) were observed between DGI total and core food scores with weight and WC, and between DGI core food score and BF (except where the outcome was MPQ scored at the matching pain site) (Supplementary Table S3). There was no mediation via weight, WC, or BF for any measures of diet quality and pain outcomes at baseline. There were significant positive direct effects between DGI core food scores and SF36-BPS (path c’) when accounting for each adiposity mediator (weight, WC, and BF). The positive relationship indicated higher intake of core foods was associated with higher SF36-BPS scores (less bodily pain). The proportion of the total effect accounted for by the indirect effect between DGI total scores and SF36-BPS (RIT) was < 30%, and < 10% for core food scores.

### Change mediation outcomes

#### Adiposity as the mediator

Changes in adiposity did not mediate the association between improvements in diet quality and pain outcomes (Table 3). Significant mediator-outcome relationships (path b) between change in weight with presence of CMP and SF36-BPS (for DGI non-core food scores as the exposure) suggested a reduction in weight was associated with a decrease in pain. Similar significant negative relationships were observed between reductions in WC and presence of CMP (in models with DGI total and non-cores scores as exposure), and between BF and SF36-BPS. For changes in SF36-BPS, although the indirect effects did not reach significance, the proportion mediated by changes in adiposity for DGI total scores ranged from 24% (for WC), to 79% (for BF).

**Table 3:** Mediation by adiposity. Direct and indirect relationships for changes in DGI total and sub scores, and changes in adiposity (weight, WC, and BF) on pain outcomes

Analysis	Path a intervention-mediator coefficient (95% CI)	Path b mediator-outcome coefficient (95% CI)	Path c’ direct effect coefficient (95% CI)	Path (a, b) indirect effect coefficient (95% CI)	Proportion mediated (RIT)^a^
Diet quality > ∆ weight (kg) > CMP (n = 104)					
∆ DGI total	− 0.021 (-0.050, 0.008)	− **0.220 (**− **0.425, **− **0.016)**	− 0.016 (− 0.054, 0.022)	0.005 (− 0.003, 0.012)	
∆ DGI core	− 0.067 (− 0.138, 0.004)	− **0.209 (**− **0.411, **− **0.007)**	− 0.020 (− 0.096, 0.056)	0.014 (− 0.006, 0.034)	
∆ DGI non-core	− 0.014 (− 0.051, 0.022)	− **0.211 (**− **0.410, -0.011)**	− 0.016 (− 0.061, 0.029)	0.003 (-0.005, 0.011)	
Diet quality > ∆ WC (cm) > CMP (n = 87)					
∆ DGI total	− 0.025 (− 0.072, 0.022)	− **0.157 (**− **0.305, **− **0.009)**	− 0.029 (− 0.071, 0.012)	0.003 (− 0.004, 0.012)	
∆ DGI core	− 0.080 (− 0.191, 0.031)	− 0.120 (− 0.253, 0.013)	− 0.021 (− 0.099, 0.058)	0.009 (− 0.007, 0.027)	
∆ DGI non-core	− 0.017 (-0.076, 0.041)	− **0.155 (**− **0.302, **− **0.009)**	− 0.036 (− 0.085, 0.014)	0.003 (− 0.007, 0.012)	
Diet quality > ∆ BF > CMP (n = 82)					
∆ DGI total	− 0.019 (− 0.039, 0.001)	− 0.130 (− 0.487, 0.227)	− 0.018 (− 0.056, 0.020)	0.002 (− 0.005, 0.010)	
∆ DGI core	− **0.059 (**− **0.106, -0.013)**	− 0.088 (− 0.428, 0.252)	− 0.003 (− 0.081, 0.074)	0.005 (− 0.015, 0.026)	
∆ DGI non-core	− 0.013 (− 0.038, 0.012)	− 0.132 (− 0.489, 0.225)	− 0.027 (− 0.074, 0.020)	0.002 (− 0.004, 0.008)	
Diet quality > ∆ weight (kg) > ∆ SF36-BPS (n = 104)					
∆ DGI total	− 0.021 (− 0.050, 0.008)	− 0.972 (− 1.948, 0.003)	0.018 (− 0.136, 0.172)	0.020 (− 0.015, 0.055)	53%
∆ DGI core	− 0.067 (− 0.138, 0.004)	− 0.911 (− 1.886, 0.064)	0.174 (− 0.206, 0.553)	0.061 (− 0.031, 0.153)	26%
∆ DGI non-core	− 0.014 (− 0.051, 0.022)	− **1.001 (**− **1.970, **− **0.032)**	− 0.015 (− 0.202, 0.172)	0.014 (− 0.024, 0.053)	
Diet quality > ∆ WC (cm) > ∆ SF36-BPS (n = 87)					
∆ DGI total	− 0.025 (− 0.072, 0.022)	− 0.613 (− 1.34, 0.115)	0.048 (− 0.121, 0.217)	0.015 (− 0.019, 0.049)	24%
∆ DGI core	− 0.080 (− 0.191, 0.031)	− 0.554 (− 1.275, 0.167)	0.320 (− 0.082, 0.721)	0.044 (− 0.040, 0.128)	12%
∆ DGI non-core	− 0.017 (− 0.076, 0.041)	− 0.645 (-1.370, 0.081)	− 0.011 (− 0.221, 0.199)	0.011 (− 0.029, 0.051)	
Diet quality > ∆ BF > ∆ SF36-BPS (n = 82)					
∆ DGI total	− 0.019 (− 0.039, 0.001)	− **2.369 (**− **4.215, **− **0.524)**	0.012 (− 0.160, 0.184)	0.044 (− 0.014, 0.103)	79%
∆ DGI core	− **0.059 (**− **0.106, **− **0.013)**	− **2.168 (**− **4.036, **− **0.299)**	0.192 (− 0.226, 0.609)	0.128 (− 0.022, 0.278)	40%
∆ DGI non-core	− 0.013 (− 0.038, 0.012)	− **2.421 (**− **4.241, **− **0.601)**	-0.031 (-0.242, 0.181)	0.032 (-0.034, 0.098)	
Diet quality > ∆ weight (kg) > ∆ MPQ (worst site, n = 46)					
∆ DGI total	− 0.052 (− 0.104, 0.000)	− 0.070 (− 0.429, 0.288)	− 0.024 (− 0.096, 0.046)	0.003 (− 0.015, 0.023)	
∆ DGI core	− 0.114 (− 0.228, 0.000)	− 0.026 (− 0.383, 0.332)	0.011 (− 0.144, 0.166)	0.003 (− 0.038, 0.044)	21%
∆ DGI non-core	− 0.042 (− 0.108, 0.024)	− 0.066 (− 0.415, 0.282)	− 0.038 (− 0.123, 0.046)	0.003 (− 0.013, 0.018)	
Diet quality > ∆ WC (cm) > ∆ MPQ (worst site, n = 39)					
∆ DGI total	− **0.120 (**− **0.200, **− **0.040)**	− 0.072 (− 0.305, 0.160)	− 0.057 (− 0.126, 0.013)	0.009 (− 0.020, 0.037)	
∆ DGI core	− 0.134 (− 0.329, 0.060)	0.009 (− 0.209, 0.227)	− 0.020 (− 0.171, 0.131)	− 0.001 (− 0.031, 0.028)	6%
∆ DGI non-core	− **0.141 (**− **0.241, **− **0.041)**	− 0.079 (− 0.309, 0.150)	− 0.076 (− 0.159, 0.007)	0.011 (− 0.022, 0.044)	
Diet quality > ∆ BF > ∆ MPQ (worst site, n = 37)					
∆ DGI total	− 0.029 (− 0.062, 0.005)	0.041 (− 0.575, 0.657)	− 0.050 (− 0.115, 0.016)	− 0.001 (− 0.019, 0.016)	2%
∆ DGI core	− 0.053 (− 0.130, 0.022)	0.160 (− 0.468, 0.789)	− 0.006 (− 0.161, 0.150)	− 0.009 (− 0.045, 0.027)	59%
∆ DGI non-core	− 0.028 (− 0.070, 0.014)	0.050 (− 0.549, 0.650)	− 0.072 (− 0.152, 0.006)	− 0.001 (− 0.019, 0.016)	2%
Diet quality > ∆ weight (kg) > ∆ MPQ (matched site, n = 43)					
∆ DGI total	− 0.039 (− 0.093, 0.015)	− 0.075 (− 0.487, 0.337)	− 0.030 (− 0.109, 0.049)	0.003 (− 0.014, 0.019)	
∆ DGI core	− 0.112 (− 0.228, 0.003)	− 0.069 (− 0.490, 0.351)	− 0.045 (− 0.223, 0.133)	0.008 (− 0.040, 0.056)	
∆ DGI non-core	− 0.024 (− 0.095, 0.046)	− 0.055 (− 0.460, 0.349)	− 0.033 (− 0.132, 0.066)	0.001 (− 0.009, 0.012)	
Diet quality > ∆ WC (cm) > ∆ MPQ (matched site, n = 36)					
∆ DGI total	− **0.099 (**− **0.178, **− **0.019)**	− 0.165 (− 0.402, 0.072)	− **0.085 (**− **0.151, **− **0.019)**	0.016 (− 0.011, 0.043)	
∆ DGI core	− 0.141 (− 0.333, 0.050)	− 0.095 (− 0.324, 0.134)	− **0.158 (**− **0.314, **− **0.002)**	0.013 (− 0.024, 0.050)	
∆ DGI non-core	− **0.119 (**− **0.223, 0.016)**	− 0.137 (− 0.380, 0.105)	− **0.087 (**− **0.172, -0.001)**	0.016 (− 0.016, 0.049)	
Diet quality > ∆ BF > ∆ MPQ (matched site, n = 34)					
∆ DGI total	− 0.018 (− 0.052, 0.016)	0.083 (− 0.520, 0.687)	− **0.073 (**− **0.135, **− **0.012)**	− 0.002 (− 0.013, 0.010)	2%
∆ DGI core	− 0.050 (− 0.127, 0.028)	0. 096 (− 0.535, 0.727)	− 0.131 (− 0.289, 0.027)	− 0.005, (− 0.037, 0.027)	4%
∆ DGI non-core	− 0.015 (− 0.060, 0.030)	0.134 (− 0.472, 0.741)	− **0.086 (**− **0.167, **− **0.005)**	− 0.002 (− 0.013, 0.009)	2%

Structural equation modelling (SEM) regression coefficients (95% CI) for exposure-mediator (path a), mediator-outcome (path b), direct (path c’) and indirect (path a, b) relationships. Covariates in the model; age, sex, and baseline values. ^**a**^ Ratio of indirect to total effect (RIT), not provided when direct and indirect in opposite directions. Bold values denote statistical significance at the p < 0.05 level.

BF, percent body fat; BMI, body mass index; CI, confidence interval; DGI, dietary guideline index; MPQ, McGill Pain Questionnaire; SF36-BPS, Short Form-36 bodily pain scale; WC, waist circumference.

Direct effects were observed between improvements in DGI total or non-core scores and reductions in pain severity (MPQ scored at the same pain site), accounting for changes in the mediators, WC and BF. Additionally, there was a direct effect between DGI core scores and MPQ (site matched) accounting for reductions in the mediator,WC.

## Discussion

We investigated the effects of a 3-month energy-restricted diet on diet quality, adiposity, and pain, exploring whether measures of adiposity mediated the relationship between diet quality and pain, before and after the intervention. At baseline, diet quality of most participants was poor and musculoskeletal pain was common, with close to half presenting with CMP, mostly involving weight bearing joints. At 3-months, significant weight loss was achieved, with improvements in overall diet quality, related to beneficial changes in the intake and quality of core and non-core foods. Significant reductions in presence of CMP, and pain severity were observed, along with improvements in quality of life related to bodily pain. Mediation analyses determined that a better-quality diet at baseline (pre-intervention), with higher scores for intake of core foods, was directly related with less bodily pain (SF36-BPS). By 3-months, higher overall diet quality was directly related to reduced pain severity, but only when comparing the same pain site. However, despite the initial relationships between lower diet quality scores and higher levels of adiposity at baseline, none of the adiposity measures mediated a relationship between diet quality and pain at baseline or after the intervention.

Diet quality is not often prioritised in weight-loss interventions [28]. This was highlighted in a recent systematic review where only half of the 18 included weight loss interventions reported concomitant improvements in diet quality, assessed by the HEI [28]. The current intervention was designed to restrict energy intake by promoting dietary changes in line with the Australian Dietary Guidelines [42]. As a result, participants not only reduced their total energy intake, but also increased their consumption of fruit, vegetables and lean meats and alternatives. Additional improvements were seen in the quality of foods consumed, reflected in improved DGI scores for choosing reduced fat dairy and whole grains, as well as in a reduction in alcohol intake. The most substantial change was a reduction in the intake in discretionary foods, resulting in improvement in DGI scores for non-core foods. These findings are important in the context of which food groups to focus on in future interventions to achieve both weight loss and improve overall diet quality.

Our baseline findings of significant direct effects between higher DGI core food scores and less bodily pain (SF36-BPS) are consistent with the limited studies that have used mediation models to explore the relationship between diet quality and pain. Better diet quality, assessed by the Healthy Eating Index (HEI) and based on adherence to the Dietary Guidelines for Americans, mediated the relationship between body fat and bodily pain (assessed via SF36-BPS) in a cross-sectional analysis of 100 adults, suggesting that the relationship between body fat and bodily pain is (at least partially) explained by diet quality [56]. Similarly, a direct relationship was observed between the intake of DGI core food scores on bodily pain, irrespective of adiposity levels, in a cross-sectional analysis of a community sample of Australian adults [57].

It is well accepted that weight loss (achieved via dietary or surgical intervention) is beneficial in managing CMP, particularly in weight bearing joints [24, 58, 59]. Systematic reviews evaluating dietary interventions for pain management have included dietary approaches that may or may not lead to weight loss [34, 35, 60, 61], making it difficult to determine the independent effects of diet alone on pain. However, these reviews conclude that altering overall diet has the greatest potential to benefit chronic pain [34–36, 60, 61]. While our mediation analysis did not establish that the improvements in all pain outcomes could be explained by changes in diet quality, there was some evidence for increases in diet quality having direct effects on improvements in pain severity, when accounting for a reduced waist circumference, and reductions in body fat.

Improvements in pain measures were not mediated through reductions in weight, WC, or BF. However, within the mediation models, reductions in weight were associated with reductions in presence of CMP, and reductions in body fat were associated with a decrease in bodily pain (SF36-BPS). These findings suggest that a lessening of body fat may reduce pain and improve pain related quality of life, consistent with previous studies [22–24]. However, there was no relationship with improvements in pain severity, suggesting there may be confounding factors not considered in the study models that influence an individual’s pain experience beyond reductions in adiposity. Exercise components were not part of the prescribed energy restriction in this study, and participants reported no changes in physical activity over time [41].

### Strengths and limitations

Several limitations need to be considered. The primary study governed the sample size of the current study, and the lack of a weight stable group constrained the analyses and interpretation of outcomes as a function of time. With subjective symptoms such as pain, the placebo response is relevant. Even if the primary objectives of the intervention study were not pain outcomes, the dietary intervention for weight loss may have produced a positive effect on self-perceived control, and subsequent subjective experience of pain [62].

Considering the aim of the study and prior theory, a strength of the study was the use of mediation analysis to evaluate relationships between diet quality, adiposity and pain outcomes. This approach examined not only the direct impact of diet quality on pain, but also indirect pathways, that enhance our understanding of the intervention’s effects. Nevertheless, capacity of the DEXA restricted eligibility for the study to an upper BMI limit of 34.9 kg/m^2^. As such, the BMI range may not have been high enough to see an effect or to establish inflammatory patterns [63, 64]. Furthermore, Covid-19 enforced restrictions prevented some body composition measures at 3-months. Additionally, the study did not capture clinically relevant inflammatory biomarkers (such as tumour necrosis factor-alpha (TNF-α), and Interleukin (IL) 1 and 6) that potentially mediate pain outcomes [65–68].

The use of validated methods to capture and assess pain presence and severity was a key strength, but how pain was captured may have influenced findings. SF36-BPS measured acute and chronic bodily pain in all participants, whereas pain severity was only captured in participants reporting CMP. Further, participants were asked to report any bodily pain in the previous 24 h so CMP may not have been identified if a participant had a pain-free day preceding assessment. Considering the study population were not a pain population, the relatively low pain levels assessed via MPQ may have contributed to the strength of observed associations.

## Conclusion

Although diet quality improved and pain levels reduced with intervention, we found that improvements in diet quality did not consistently lead to reductions in pain in the mediation models. Further, reductions in adiposity did not mediate the effect of the intervention on pain. However, there was some evidence that improvements in diet quality directly influenced pain severity when accounting for reductions in waist circumference and body fat.

Accordingly, results from the mediation models were inconclusive in determining whether improved diet quality or reductions in adiposity were responsible for the improvements in pain. Future mediation analyses assessing the relationship between improvements in diet quality and pain outcomes should comprise of larger sample sizes to include a control group, be conducted in participants across a greater BMI range, as well as in specific chronic pain conditions. Nevertheless, this study contributes to the limited literature, and advances our understanding, of the need for lifestyle interventions for pain management that focus on enhancing diet quality and reducing adiposity.

## Supplementary Information

Below is the link to the electronic supplementary material.

Supplementary Material 1.

Supplementary Material 2.

Supplementary Material 3.

## Data Availability

Datasets are available on request.
