# Supplementary material for: Exploring the role of diet quality and adiposity in the pain experience: a mediation analysis
Source: Eur J Nutr. 2025 Aug 23;64(6):266. doi: 10.1007/s00394-025-03772-0 (PMC12374914; doi:10.1007/s00394-025-03772-0)
Supplement: Supplementary file 1 — Supplementary Material 1. [file 394_2025_3772_MOESM1_ESM.docx]

# ****Title:** Exploring the role of diet quality and adiposity in the pain experience: A mediation analysis**

Journal: European Journal of Nutrition

Authors: Susan J Ward ^1,2^, Alison M Coates ^1,3^, Sharayah Carter ^1,3,4^, Katherine L Baldock ^3^, Ty E Stanford ^1,3^, Carolyn Berryman ^3,5^, Tasha R Stanton ^3,5,6^, Jonathan D Buckley ^1,3^, Alison M Hill ^1,2*^

^1^ Alliance for Research in Exercise, Nutrition and Activity (ARENA), University of South Australia, Adelaide, South Australia, Australia

^2^ Clinical and Health Sciences, University of South Australia, Adelaide, South Australia, Australia

^3^ Allied Health and Human Performance, University of South Australia, Adelaide, South Australia, Australia

^4^ School of Health and Biomedical Sciences, Royal Melbourne Institute of Technology (RMIT University), Melbourne, Victoria, Australia

^5^ Innovation, IMPlementation And Clinical Translation (IIMPACT), University of South Australia, Adelaide, South Australia, Australia

^6^ Persistent Pain Research Group, Hopwood Centre for Neurobiology, South Australian Health and Medical Research Institute (SAHMRI) Adelaide, South Australia, Australia

Corresponding author: alison.hill@unisa.edu.au

**Supplementary Table S1** Components and scoring for the Dietary Guideline Index ^a^

| Dietary Guideline | Indicator & description | Criteria for minimum score | Criteria for maximum score | Component score |
| --- | --- | --- | --- | --- |
| Core food components: adequate intake | | | | |
| 1. Enjoy a wide variety of nutritious foods from each of the 5 core food groups every day. | **Food variety:**  proportion of food types from each of the 5 core food groups consumed at least once per week. | 0% | 100% | 10 |
| 2. Plenty of vegetables, legumes/beans | **Total vegetable intake:**  servings of vegetables per day | 0 serves | 19 - 50 years:  M ≥ 6, F ≥ 5  51 – 70 years:  M ≥ 5.5, F ≥ 5  > 70 years:  M ≥ 5, F ≥ 5 | 10 |
| 3. Fruit | **Total fruit intake:**  servings of fruit per day | 0 serves | M $\geq$ 2 serves  F $\geq$ 2 serves | 10 |
| 4. Grain (cereal) foods | **Total grain intake:**  servings of grains per day | 0 serves | 19 - 50 years:  M ≥ 6, F ≥ 6  51 – 70 years:  M ≥ 6, F ≥ 4  > 70 years:  M ≥ 4.5, F ≥ 3 | 5 |
|  | **4a Mostly wholegrain or high fibre cereals:**  serves of wholegrain as a proportion of total grains | <50% | >50% | 5 |
| 5. Lean meat and poultry, fish, eggs, nuts and seeds, and legumes/beans | **Total lean meat and alternatives:**  servings per day (excludes processed meat) | 0 serves | 19 - 50 years:  M ≥ 3, F ≥ 2.5  51 – 70 years:  M ≥ 2.5, F ≥ 2  > 70 years:  M ≥ 2.5 serves,  F ≥ 2 serves | 10 |
| 6. Milk, yoghurt, cheese  and/or their alternatives | **Total dairy and alternatives:**  servings per day | 0 serves | 19 - 50 years:  M ≥ 2.5, F ≥ 2.5  51 – 70 years:  M ≥ 2.5, F ≥ 4  > 70 years:  M ≥ 3.5, F ≥ 4 | 5 |
|  | **6a. Choose reduced-fat milk:** type of milk usually consumed | <100% | >100% | 5 |
| 7. Drink plenty of water | **Total beverage intake:** servings per day | 0 serves | M $\geq$ 10 serves  F $\geq$ 8 serves | 5 |
|  | **7a.** Proportion of water consumed relative to total ml of beverages | 0% | >50% | 5 |
| Non-core food components: moderate or limit intake | | | | |
| 8. Small allowance of unsaturated oils, fats, or spreads | **Unsaturated spreads or oils:**  serves per day | 50 – 70 years:  M <1 & >4 serves  F <1 & >2 serves  > 70 years:  M & F  <1 & >2 serves | 50 – 70 years:  M 1-4 serves  F 1-2 serves  > 70 years:  M & F 1-2 serves | 10 |
| 9. Limit intake of foods and drinks containing saturated fat, added salt, and added sugars | **Limit discretionary foods:** servings of discretionary foods per day | 19 – 50 years:  M $\geq$ 3, F $\geq$ 2.5  51 – 70 years:  M $\geq$ 2.5, F $\geq$ 2.5  > 70 years:  M $\geq$ 2.5, F $\geq$ 2 | 19 – 50 years:  M 0 - 3, F 0 - 2.5  51 – 70 years:  M 0 - 2.5, F 0 - 2.5  > 70 years:  M 0 - 2.5, F 0 - 2 | 10 |
| 10. If you choose to drink alcohol, limit intake^b^ | **Limit alcohol:**  serves per day | >1.4 serves | $\leq$ 1.4 serves | 10 |

^a^ Adapted from Ward et al [45]

^b^ NHMRC guidelines to reduce health risks from drinking alcohol (no more than 2 standard drinks (10g alcohol)/day) used for cut-offs [69]
