# Supplementary material for: Exploring the role of diet quality and adiposity in the pain experience: a mediation analysis
Source: Eur J Nutr. 2025 Aug 23;64(6):266. doi: 10.1007/s00394-025-03772-0 (PMC12374914; doi:10.1007/s00394-025-03772-0)
Supplement: Supplementary file 2 — Supplementary Material 2. [file 394_2025_3772_MOESM2_ESM.docx]

# ****Title:** Exploring the role of diet quality and adiposity in the pain experience: A mediation analysis**

Journal: European Journal of Nutrition

Authors: Susan J Ward ^1,2^, Alison M Coates ^1,3^, Sharayah Carter ^1,3,4^, Katherine L Baldock ^3^, Ty E Stanford ^1,3^, Carolyn Berryman ^3,5^, Tasha R Stanton ^3,5,6^, Jonathan D Buckley ^1,3^, Alison M Hill ^1,2*^

^1^ Alliance for Research in Exercise, Nutrition and Activity (ARENA), University of South Australia, Adelaide, South Australia, Australia

^2^ Clinical and Health Sciences, University of South Australia, Adelaide, South Australia, Australia

^3^ Allied Health and Human Performance, University of South Australia, Adelaide, South Australia, Australia

^4^ School of Health and Biomedical Sciences, Royal Melbourne Institute of Technology (RMIT University), Melbourne, Victoria, Australia

^5^ Innovation, IMPlementation And Clinical Translation (IIMPACT), University of South Australia, Adelaide, South Australia, Australia

^6^ Persistent Pain Research Group, Hopwood Centre for Neurobiology, South Australian Health and Medical Research Institute (SAHMRI) Adelaide, South Australia, Australia

Corresponding author: alison.hill@unisa.edu.au

**Supplementary Table S2** Baseline associations between variables for inclusion in the mediation

|  | **Diet Quality** | | | **Pain** | | | | **Adiposity** | | |
| --- | --- | --- | --- | --- | --- | --- | --- | --- | --- | --- |
|  | **DGI Total**  **(0-120)** | **DGI Core**  **(0-70)** | **DGI**  **Non-core**  **(0-50)** | **Presence of**  **CMP**  **(yes/no)** | **SF36-BPS**  **(0-100)** | **MPQ Worst site**  **(0-45)** | **MPQ Matched site (0-45)** | **Weight (kg)** | **WC (cm)** | **BF (%)** |
| **n** | **134** | **134** | **134** | **134** | **134** | **63** | **45** | **134** | **134** | **134** |
| **Age**^a^ | 0.03  (-0.14, 0.20) | 0.17  (-0.00, 0.33) | -0.09  (-0.25, 0.08) | 0.07  (-0.10, 0.24) | -0.15  (-0.31, 0.02) | -0.10  (-0.34, 0.16) | -0.15  (-0.41, 0.14) | -0.14  (-0.30, 0.03) | 0.20  (-0.05, 0.28) | 0.11  (-0.06, 0.28) |
| **Sex**^b,c^ | -0.003  (-0.17, 0.17) | -0.22  (-0.38, -0.05) | 0.12  (-0.05, 0.28) | 0.79  (V = 0.08) | 0.16  (-0.01, 0.32) | -0.08  (-0.32, 0.17) | -0.02  (-0.31, 0.28) | 0.54  (0.41, 0.65) | 0.46  (0.31, 0.58) | -0.74  (-0.81, -0.65) |
| **SEIFA**^a,c^ | 0.11  (-0.051, 0.2) | 0.12  (-0.06, 0.28) | 0.12  (-0.05, 0.29) | 9.86  (V = 0.27) | 0.03  (-0.14, 0.20) | -0.20  (-0.43, 0.05) | -0.26  (-0.46, 0.08) | -0.12  (-0.29, 0.05) | -0.12  (-0.29, 0.05) | 0.04  (-0.13, 0.21) |
| **Energy intake (kJ/day)**^a^ | -0.11  (-0.28, 0.06) | 0.23  (0.06, 0.38) | -0.27  (-0.43, -0.11) | 0.12  (-0.05, 0.29) | -0.09  (-0.26, 0.08) | 0.22  (-0.03, 0.45) | 0.11  (-0.20, 0.36) | 0.31  (0.14, 0.46) | 0.18  (0.01, 0.34) | -0.08  (-0.24, 0.10) |

Relationships assessed via ^a^ Spearman-rank correlation with 95% CI estimations based on formula proposed by Bonett and Wright, ^b^ Point serial correlation, ^c^ Χ^2^ Chi-square test of independence, with Cramer’s V for association with CMP

Abbreviations: BF, percent body fat; CMP, chronic musculoskeletal pain; CI, confidence interval; DGI, dietary guideline index; MPQ, McGill Pain Questionnaire; SF36-BPS, Short Form-36 bodily pain scale; WC, waist circumference
