# Supplementary material for: Exploring the role of diet quality and adiposity in the pain experience: a mediation analysis
Source: Eur J Nutr. 2025 Aug 23;64(6):266. doi: 10.1007/s00394-025-03772-0 (PMC12374914; doi:10.1007/s00394-025-03772-0)
Supplement: Supplementary file 3 — Supplementary Material 3. [file 394_2025_3772_MOESM3_ESM.docx]

# ****Title:** Exploring the role of diet quality and adiposity in the pain experience: A mediation analysis**

Journal: European Journal of Nutrition

Authors: Susan J Ward ^1,2^, Alison M Coates ^1,3^, Sharayah Carter ^1,3,4^, Katherine L Baldock ^3^, Ty E Stanford ^1,3^, Carolyn Berryman ^3,5^, Tasha R Stanton ^3,5,6^, Jonathan D Buckley ^1,3^, Alison M Hill ^1,2*^

^1^ Alliance for Research in Exercise, Nutrition and Activity (ARENA), University of South Australia, Adelaide, South Australia, Australia

^2^ Clinical and Health Sciences, University of South Australia, Adelaide, South Australia, Australia

^3^ Allied Health and Human Performance, University of South Australia, Adelaide, South Australia, Australia

^4^ School of Health and Biomedical Sciences, Royal Melbourne Institute of Technology (RMIT University), Melbourne, Victoria, Australia

^5^ Innovation, IMPlementation And Clinical Translation (IIMPACT), University of South Australia, Adelaide, South Australia, Australia

^6^ Persistent Pain Research Group, Hopwood Centre for Neurobiology, South Australian Health and Medical Research Institute (SAHMRI) Adelaide, South Australia, Australia

Corresponding author: alison.hill@unisa.edu.au

**Supplementary Table S3** Mediation by adiposity. Direct and indirect relationships for DGI total and sub scores, weight, WC, and BF, on pain outcomes.

| **Analysis** | **Path a**  **Exposure-mediator Coefficient (95% CI)** | | **Path b**  **Mediator-outcome Coefficient (95% CI)** | **Path c’**  **Direct Effect**  **Coefficient (95% CI)** | **Path a, b**  **Indirect Effect**  **Coefficient (95% CI)** | **Proportion Mediated**  **(RIT)^a^** |
| --- | --- | --- | --- | --- | --- | --- |
| **DGI > Weight (kg) > CMP (n = 134)** | | | | | | |
| DGI total | | **-0.** **128 (-0.227, -0.029)** | 0.014 (-0.025, 0.054) | 0.002 (-0.022, 0.025) | -0.002 (-0.007, 0.003) |  |
| DGI core | | **-0.282 (-0.510, -0.053)** | 0.017 (-0.022, 0.056) | 0.020 (-0.033, 0.074) | -0.005 (-0.017, 0.007) |  |
| DGI non-core | | -0.121 (-0.249, 0.006) | 0.013 (-0.025, 0.052) | -0.004 (-0.033, 0.026) | -0.002 (-0.007, 0.003) | 31% |
| **DGI > WC (cm) > CMP (n = 134)** | | | | | | |
| DGI total | | **-0.093 (-0.179, -0.008)** | 0.012 (-0.033, 0.057) | 0.001 (-0.022, 0.024) | -0.001 (-0.005, 0.003) |  |
| DGI core | | **-0.252 (-0.448, -0.056)** | 0.016 (-0.030, 0.061) | 0.019 (-0.034, 0.073) | -0.004 (-0.016, 0.008) |  |
| DGI non-core | | -0.074 (-0.184, 0.036) | 0.011 (-0.033, 0.056) | -0.004 (-0.034, 0.025) | -0.001 (-0.004, 0.003) | 16% |
| **DGI > BF > CMP (n = 134)** | | | | | | |
| DGI total | | -0.010 (-0.056, 0.036) | -0.040 (-0.124, 0.043) | -0.001 (-0.024, 0.022) | 0.0004 (-0.002, 0.002) |  |
| DGI core | | **-0.105 (-0.210, -0.0002)** | -0.037 (-0.122, 0.047) | 0.011 (-0.042, 0.064) | 0.004 (-0.006, 0.014) | 26% |
| DGI non-core | | 0.016 (-0.042, 0.075) | -0.040 (-0.124, 0.044) | -0.005 (-0.034, 0.025) | -0.001 (-0.003, 0.002) | 12% |
| **DGI > Weight (kg) > SF36-BPS (n = 134)** | | | | | | |
| DGI total | | **-0.128 (-0.227, -0.029)** | -0.310 (-0.706, 0.085) | 0.091 (-0.145, 0.328) | 0.040 (-0.019, 0.099) | 30% |
| DGI core | | **-0.282 (-0.510, -0.053)** | -0.250 (-0.638, 0.138) | **0.628 (0.093, 1.162)** | 0.070 (-0.053, 0.194) | 10% |
| DGI non-core | | -0.121 (-0.249, 0.006) | -0.352 (-0.744, 0.040) | -0.043 (-0.342, 0.255) | 0.043 (-0.023, 0.108) |  |
| **DGI > WC (cm) > SF36-BPS (n = 134)** | | | | | | |
| DGI total | | **-0.093 (-0.179, -0.008)** | -0.187 (-0.647, 0.274) | 0.114 (-0.123, 0.350) | 0.017 (-0.028, 0.063) | 13% |
| DGI core | | **-0.252 (-0.448, -0.056)** | -0.106 (-0.561, 0.349) | **0.672 (0.133, 1.210)** | 0.027 (-0.090, 0.143) | 4% |
| DGI non-core | | -0.074 (-0.184, 0.036) | -0.230 (-0.687, 0.227) | -0.018 (-0.317, 0.282) | 0.017 (-0.025, 0.059) |  |
| **DGI > BF > SF36-BPS (n = 134)** | | | | | | |
| DGI total | | -0.010 (-0.056, 0.036) | -0.180 (-1.036, 0.676) | 0.129 (-0.104, 0.362) | 0.002 (-0.010, 0.014) | 1% |
| DGI core | | **0.105 (-0.210, -0.000)** | -0.011 (-0.862, 0.839) | **0.697 (0.163, 1.231)** | 0.001 (-0.088, 0.091) | 0.2% |
| DGI non-core | | 0.016 (-0.042, 0.075) | -0.197 (-1.057, 0.662) | 0.002 (-0.296, 0.301) | -0.003 (-0.021, 0.015) |  |
| **DGI > Weight (kg) > MPQ (Worst site, n = 63)** | | | | | | |
| DGI total | | **-0.277 (-0.421, -0.134)** | 0.030 (-0.153, 0.214) | -0.062 (-0.180, 0.056) | -0.008 (-0.060, 0.043) | 12% |
| DGI core | | **-0.456 (-0.752, -0.160)** | 0.020 (-0.154, 0.196) | -0.183 (-0.408, 0.042) | -0.009 (-0.090, 0.071) | 5% |
| DGI non-core | | **-0.257 (-0.447, -0.067)** | 0.065 (-0.111, 0.241) | -0.018 (-0.160, 0.125) | -0.017 (-0.063, 0.030) | 49% |
| **DGI > WC (cm) > MPQ (Worst site, n = 63)** | | | | | | |
| DGI total | | **-0.271 (-0.406, -0.135)** | 0.007 (-0.188, 0.201) | -0.068 (-0.188, 0.051) | -0.002 (-0.054, 0.051) | 3% |
| DGI core | | **-0.454 (-0.733, -0.174)** | -0.003 (-0.189, 0.183) | -0.194 (-0.420, 0.033) | 0.001 (-0.083, 0.086) |  |
| DGI non-core | | **-0.248 (-0.428, -0.068)** | 0.047 (-0.139, 0.233) | -0.023 (-0.166, 0.121) | -0.012 (-0.058, 0.035) | 34% |
| **DGI > BF> MPQ (Worst site, n = 63)** | | | | | | |
| DGI total | | -0.065 (-0.136, 0.006) | 0.103 (-0.267, 0.473) | -0.063 (-0.173, 0.046) | -0.007 (-0.032, 0.018) | 10% |
| DGI core | | **-0.149 (-0.290, -0.009)** | 0.072 (-0.298, 0.441) | -0.182 (-0.399, 0.035) | -0.011 (-0.067, 0.045) | 6% |
| DGI non-core | | -0.043 (-0.134, 0.048) | 0.142 (-0.225, 0.509) | -0.028 (-0.164, 0.108) | -0.006 (-0.026, 0.014) | 18% |
| **DGI > Weight (kg) > MPQ (Matched site, n = 45)** | | | | | | |
| DGI total | | **-0.324 (-0.503, -0.144)** | -0.032 (-0.256, 0.191) | -0.102 (-0.257, 0.053) | 0.010 (-0.062, 0.083) |  |
| DGI core | | **-0.** **520 (-0.** **840, -0.200)** | 0.018 (-0.204, 0.241) | -0.050 (-0.320, 0.220) | -0.009 (-0.125, 0.106) | 16% |
| DGI non-core | | **-0.260 (-0.515, -0.006)** | -0.003 (-0.210, 0.203) | -0.123 (-0.310, 0.064) | 0.001 (-0.053, 0.055) |  |
| **DGI > WC (cm) > MPQ (Matched site, n = 45)** | | | | | | |
| DGI total | | **-0.255 (-0.436, -0.074)** | -0.128 (-0.347, 0.091) | -0.124 (-0.270, 0.023) | 0.032 (-0.028, 0.092) |  |
| DGI core | | **-0.429 (-0.747, -0.112)** | -0.088 (-0.310, 0.134) | -0.097 (-0.357, 0.163) | 0.038 (-0.062, 0.138) |  |
| DGI non-core | | -0.194 (-0.444, 0.056) | -0.093 (-0.302, 0.115) | -0.140 (-0.323, 0.042) | 0.018 (-0.029, 0.065) |  |
| **DGI > BF > MPQ (Matched site, n = 45)** | | | | | | |
| DGI total | | -0.038 (-0.107, 0.032) | -0.390 (-0.955, 0.176) | -0.106 (-0.242, 0.031) | 0.015 (-0.020, 0.049) |  |
| DGI core | | -0.114 (-0.232, 0.005) | -0.388 (-0.979, 0.203) | -0.103 (-0.352, 0.145) | 0.044 (-0.037, 0.125) |  |
| DGI non-core | | -0.0003 (-0.092, 0.092) | -0.322 (-0.884, 0.240) | -0.122 (-0.300, 0.055) | 0.0001 (-0.030, 0.030) |  |

Structural equation modelling (SEM) regression coefficients (95% CI) for baseline exposure-mediator (path a), mediator-outcome (path b), direct (path c’) and indirect (path a, b) relationships. Covariates in the model; age, sex, and baseline EI. **^a^** Ratio of indirect to total effect (RIT), not provided when direct and indirect in opposite directions. Bold values denote statistical significance at the p < 0.05 level.

Abbreviations: BF, percent body fat; BMI, body mass index; CI, confidence interval; DGI, dietary guideline index; EI, energy intake; MPQ, McGill Pain Questionnaire; SF36-BPS, Short Form-36 bodily pain scale; WC, waist circumference
